# L‐Dex, arm volume, and symptom trajectories 24 months after breast cancer surgery

**DOI:** 10.1002/cam4.3188

**Published:** 2020-06-01

**Authors:** Sheila H. Ridner, Chirag Shah, John Boyages, Louise Koelmeyer, Nicolas Ajkay, Sarah M. DeSnyder, Sarah A. McLaughlin, Mary S. Dietrich

**Affiliations:** ^1^ Vanderbilt University School of Nursing Nashville TN USA; ^2^ Department of Radiation Oncology Taussig Cancer Institute Cleveland Clinic Cleveland OH USA; ^3^ Australian Lymphoedema Education, Research, and Treatment (ALERT) Program Faculty of Medicine & Health Sciences Macquarie University Sydney NSW Australia; ^4^ ALERT Program Faculty of Medicine & Health Sciences Macquarie University Sydney NSW Australia; ^5^ Breast Surgical Oncology University of Louisville Louisville KY USA; ^6^ Department of Breast Surgical Oncology The University of Texas MD Anderson Cancer Center Houston TX USA; ^7^ Surgery Mayo Clinic Jacksonville FL USA; ^8^ Vanderbilt University School of Nursing and Vanderbilt University School of Medicine Nashville TN USA

**Keywords:** biomarkers, breast cancer, quality of life, survival

## Abstract

**Purpose:**

Study objectives were to examine: (a) biomarker trajectories (change from presurgical baseline values of Lymphedema index (L‐Dex) units and arm volume difference) and symptom cluster scores 24 months after breast cancer surgery and (b) associations of these objective biomarkers and symptom cluster scores. Patient/treatment characteristics influencing trajectories were also evaluated.

**Methods:**

A secondary analysis of data from the published interim analysis of a randomized parent study was undertaken using trajectory analysis. Five hundred and eight participants included in the prior analysis with 24 months of postsurgical follow‐up were initially measured with bioelectric impedance spectroscopy (BIS) and tape measure (TM) and completed self‐report measures. Patients were reassessed postsurgery for continuing eligibility and then randomized to either BIS or TM groups and measured along with self‐report data at regular and optional* visits 3, 6,12,15*,18, 21*, and 24‐months.

**Results:**

Three subclinical trajectories were identified for each biomarker (decreasing, stable, increasing) and symptom cluster scores (stable, slight increase/decrease, increasing). Subclinical lymphedema was identified throughout the 24‐month period by each biomarker. An L‐Dex increase at 15 months in the BIS group was noted. The self‐report sets demonstrated contingency coefficients of 0.20 (LSIDS‐A soft tissue, *P* = .031) and 0.19 (FACTB+4, *P* = .044) with the L‐Dex unit change trajectories.

**Conclusions:**

These data support the need for long‐term (24 months) prospective surveillance with frequent assessments (every 3 months) at least 15 months after surgery. Statistically significant convergence of symptom cluster scores with L‐Dex unit change supports BIS as beneficial in the early identification of subclinical lymphedema.

## Graphical abstract

Long‐term (24 months) lymphedema prospective surveillance with frequent assessments (every 3 months) at least through 15‐months post‐surgery is indicated. Convergence of symptom cluster scores with L‐Dex unit change supports Bioelectric Impedance Spectroscopy as beneficial in the early identification of sub‐clinical lymphedema.

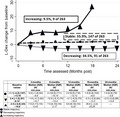

## 1 INTRODUCTION

Breast cancer‐related lymphedema (BCRL) results in quality‐of‐life altering treatment sequelae for approximately 20%‐30% of breast cancer survivors (BCS).^1^ The identification of initial increases in lymphatic fluid (subclinical lymphedema) after cancer treatment, when coupled with an early short‐term compression intervention, is thought to reduce rates of progression to clinical lymphedema.^2^, ^3^ Thus, since the publication of the Stout et al study in 2008, consensus has grown that prospective surveillance after breast cancer treatment is needed to promote early identification of subclinical lymphedema to facilitate noninvasive interventions that reduce burden and cost of clinical lymphedema.^4^, ^5^, ^6^ Prospective surveillance requires longitudinal assessments of patients’ arm status, preferably with baseline measurements before treatment commences and serially thereafter.

Biomarkers are increasingly being studied in oncology and serve as objective indicators,^7^ “…reflecting an interaction between a biological system and an environmental agent, which may be chemical, physical or biological.”^8^ In the case of lymphedema, Lymphedema index (L‐Dex) units^®^ and arm volume function as biomarkers. An L‐Dex unit change of ≥6.5 from presurgical baseline and a tape measure (TM) volume difference change of ≥5% but <10% are considered subclinical BCRL and values exceeding these thresholds for each measure represent clinical lymphedema.^9^

Little is known about the longitudinal patterns of change in L‐Dex units and arm volume difference in relation to subclinical BCRL.^1^, ^10^ This contributes to the absence of both standardized monitoring intervals and monitoring duration for BCRL prospective surveillance.^11^ In the case of lymphedema biomarkers, trajectory analysis of change in L‐Dex units and arm volume percent difference from baseline, combined with symptoms, could provide information to fill this gap.^12^, ^13^ The objectives of this secondary study were to examine: (a) the trajectories of objective biomarkers (change from presurgical baseline values of L‐Dex units and in percentage arm volume difference) and subjective symptom clusters over 24 months after surgery; and (b) associations of objective biomarkers and subjective symptom clusters. Secondarily, patient/treatment characteristics influencing the trajectories of interest were explored. It was hypothesized that longitudinal patterns in biomarkers and symptom clusters would be identified.

## 2 PATIENTS AND METHODS

### 2.1 Study setting and design

This study is comprised of data from an ongoing multi‐site, international randomized controlled trial and was conducted in accordance with the Declaration of Helsinki. Institutional Review Board approval was gained from all sites. Scientific Review Committee approval was acquired when applicable. All sites obtained informed consent prior to study enrollment.

### 2.2 Patients

Newly diagnosed patients with breast cancer (N = 1201) who met previously described inclusion criteria were enrolled in the parent study and randomized postsurgery to prospective surveillance via Bioimpedance spectroscopy (BIS) or TM.^3^ Patients underwent a lymphedema prevention intervention with a compression sleeve and gauntlet when subclinical lymphedema was identified.^3^ An interim analysis from the parent study included data from 508 participants with 12 months of postsurgical follow‐up and addressed progression to complex decongestive physiotherapy.^3^ This study includes participants from the interim analysis who have completed at least 24 months of follow‐up after date of first surgery and addresses subclinical BCRL.

### 2.3 Procedures

#### 2.3.1 Measures

##### Sociodemographic and medical characteristics

A self‐report questionnaire captured these variables for the sample that have been previously reported.^3^ Comorbidity and medication histories were obtained via self‐report and medical records.

##### Biomarkers

The ImpediMed L‐Dex^®^ U400^14^ was used to evaluate extracellular fluid following manufacturer’s procedures. Limb volume change was assessed using a nonflexible Gulick II^15^ tape according to study protocol.

##### Symptoms

The Lymphedema Symptom Intensity and Distress Survey‐Arm (LSIDS‐A) 4‐item (heavy arm, tight arm, swelling arm, hard arm) soft tissue sensation subscale^16^ and the Functional Assessment Cancer Therapy Breast +4 (FACTB+4) 4‐item (painful movement, poor range of motion, numb, stiffness) arm subscale were used in this study.^17^

### 2.4 Data Analysis

Descriptive statistics summarized baseline patient and treatment characteristics used in this study (see Tables 1 and 2). Mann‐Whitney and chi‐squared tests were used to assess study group differences.

**Table 1:** Patient‐treatment characteristics associations with L‐Dex and tape trajectories

	Patients assessed via L‐DEX (N = 263)			Patients assessed via Tape (N = 245)		
	Decreasing	Stable	Increasing	Decreasing	Stable	Increasing
	Median [IQR] (N)					
Age (y)	59^a^ [50,68], 91	58^a^ [49,68], 146	**64^b^ [56,71], 25**	61 [49,68], 65	57 [51,65], 137	58 [50,67], 41
BMI	27 [23,32], 91	28 [24,33], 147	29 [24,36], 25	29 [23,33], 65	28 [24,32], 138	30 [25,35], 41
	N Yes (% Yes) [N responses]					
Oral Steroids (N = 7)	2 (2) [91]	4 (3) [145]	1 (4) [25]	2 (3) [65]	1 (1) [138]	2 (5) [41]
NSAIDs	27 (30) [91]	**29 (20)^a^ [145]**	**12 (48)^b^ [25]**	10 (15) [66]	27 (20) [138]	11 (27) [41]
History						
Cardiovascular	**31 (34)^b^ [91]**	**73 (50)^b^ [146]**	13 (52) [25]	28 (42) [66]	57 (41) [138]	21 (51) [41]
Excretory	7 (8) [91]	6 (4) [146]	3 (12) [25]	4 (6) [66]	6 (4) [138]	1 (2) [41]
GERD	11 (69) [16]	15 (54) [28]	4 (100) [4]	12 (75) [16]	**37 (84)^a^ [44]**	**4 (40)^b^ [10]**
Respiratory	11 (12) [91]	21 (14) [146]	1 (4) [25]	7 (11) [66]	20 (15) [138]	8 (20) [40]
Regional node irradiation	15 (20) [75]	29 (22) [130]	6 (30) [20]	10 (19) [54]	27 (23) [117]	9 (29) [31]
If chemo, any Taxane	32 (94) [34]	50 (85) [59]	14 (100) [14]	27 (93) [29]	48 (92) [52]	22 (88) [25]
Axillary dissection (±SLND)	N = 87	N = 139	N = 24	N = 63	N = 133	N = 36
ALND only	8 (9)	13 (9)	3 (13)	4 (6)	12 (9)	6 (17)
SLNB only	70 (81)	115 (83)	16 (67)	49 (78)	103 (77)	24 (67)
ALND and SLNB	9 (10)	11 (8)	5 (21)	10 (16)	18 (14)	6 (17)
If SLND, number ≥6	5 (7) [70]	1 (1) [115]	0 (0) [18]	2 (4) [49]	6 (6) [103]	1 (4) [24]
Conservative mastectomy	74 (81) [91]	121 (82) [147]	17 (68) [25]	52 (79) [66]	110 (80) [138]	27 (66) [41]
Complete treatment	N = 87	N = 146	N = 25	N = 66	N = 138	N = 41
Surgery	8 (9)	9 (6)	2 (8)	6 (9)	13 (9)	5 (12)
Surgery + Radiotherapy	48 (55)	80 (55)	9 (36)	31 (47)	75 (54)	11 (27)
Surgery +Chemotherapy (Taxane)	6 (7)	6 (4)	3 (12)	8 (12)	6 (4)	5 (12)
Surgery + Chemotherapy (Not Taxane)	0 (0)	1 (1)	0 (0)	0 (0)	1 (1)	0 (0)
Surgery + Radiotherapy +Chemotherapy (Taxane)	24 (28)	42 (29)	11 (44)	19 (29)	40 (29)	17 (42)
Surgery + Radiotherapy + Chemotherapy (Not Taxane)	1 (1)	8 (6)	0 (0)	2 (3)	3 (2)	3 (7)

Bold indicates cell with statistically significant findings (Bonferroni‐corrected *P* < .05). Superscripts (^a^, ^b^) indicate specifically which trajectory patterns defined the difference.

**Table 2:** Patient‐treatment characteristics associations with self‐reported symptom trajectories

	LSIDS‐A Soft Tissue (N = 508)			FACT‐B Arm (N = 508)		
	None/Stable	Slight Increase	Increasing	None/Stable	Slight Increase	Increasing
	Median [IQR] (N)					
Age (years)	**61^a^ [52,68], 324**	55^b^ [48,65], 137	52^b^ [48,59], 44	**61^a^ [51,68], 267**	58 [50,66], 195	**53^b^ [46,60], 43**
BMI	28 [24,32], 326	28 [24,34], 137	29 [25,33], 44	28 [25,34], 269	28 [24,32], 194	30 [25,35], 44
	N Yes (% Yes) [N responses]					
Oral Steroids	10 (3) [323]	2 (1) [138]	0 (0) [44]	8 (3) [267]	3 (2) [194]	1 (2) [44]
NSAIDs	79 (24) [324]	26 (19) [138]	11 (25) [44]	55 (21) [267]	49 (25) [195]	12 (27) [44]
History						
Cardiovascular	154 (47) [325]	50 (36) [138]	19 (43) [44]	119 (44) [268]	83 (43) [195]	21 (48) [44]
Excretory	17 (5) [325]	8 (6) [138]	2 (5) [44]	18 (7) [268]	9 (5) [195]	0 (0) [44]
GERD	49 (69) [71]	24 (69) [35]	10 (83) [12]	42 (72) [58]	30 (65) [46]	11 (79) [14]
Respiratory	**29 (9)^a^ [324]**	29 (21)^b^ [138]	10 (23)^b^ [44]	**29 (11)^a^ [268]**	28 (14) [194]	**11 (25)^b^ [44]**
Regional node irradiation	**39 (14)^a^ [271]**	40 (34)^b^ [119]	17 (46)^b^ [37]	**34 (15)^a^ [231]**	46 (29)^b^ [160]	16 (44)^b^ [36]
If chemo, any Taxane	95 (88) [108]	68 (93) [73]	30 (94) [32]	83 (87) [95]	84 (94) [89]	26 (90) [29]
Axillary Dissection (+/− SLND)	N = 312	N = 128	N = 42	N = 258	N = 183	N = 41
ALND Only	11 (4)^a^	**23 (18)^b^**	**12 (29)^b^**	14 (5)^a^	19 (10)^a^	**13 (32)^b^**
SLNB Only	270 (87)^a^	**82 (64)^b^**	**25 (60)^b^**	**224 (87)^a^**	128 (70)^b^	25 (61)^b^
ALND & SLNB	31 (10)	23 (18)	5 (12)	**20 (8)^a^**	**36 (20)^b^**	3 (7)
If SLND, number ≥6	9 (3) [270]	5 (6) [82]	1 (4) [25]	5 (2) [224]	9 (7) [128]	1 (4) [25]
Conservative mastectomy	264 (81) [326]	102 (74) [138]	35 (80) [44]	224 (83) [269]	146 (75) [195]	31 (71) [44]
Complete treatment	N = 323	N = 137	N = 43	N = 268	N = 192	N = 43
Surgery	33 (10)	9 (7)	1 (2)	23 (9)	18 (9)	2 (5)
Surgery + Radiotherapy	**184 (57)^a^**	57 (42)^b^	13 (30)^b^	**153 (57)^a^**	88 (46)	**13 (30)^b^**
Surgery + Chemotherapy (Taxane)	19 (6)	11 (8)	4 (9)	13 (5)	17 (9)	4 (9)
Surgery + Chemotherapy (Not Taxane)	1 (1)	1 (1)	0 (0)	**0 (0)^a^**	1 (1)	**1 (2)^b^**
Surgery + Radiotherapy + Chemotherapy (Taxane)	**73 (23)^a^**	56 (41)^b^	24 (56)^b^	**68 (25)^a^**	64 (33)	**21 (49)^b^**
Surgery + Radiotherapy + Chemotherapy (Not Taxane)	13 (4)	3 (2)	1 (2)	11 (4)	4 (2)	2 (5)

Bold indicates cell with statistically significant findings (Bonferroni‐corrected *P* < .05); Superscripts (^a^, ^b^) indicate specifically which trajectory patterns defined the difference.

Group‐based trajectory analysis as implemented in SAS PROC TRAJ (version 9.4) was used to detect longitudinal patterns of change in L‐Dex values, change in percent arm volume difference via TM, LSIDS‐A Soft Tissue scores, and FACT‐B Arm scores beginning prior to treatment and up to 24 months thereafter.^12^ Both Bayesian information criteria and Akaike information criteria (AIC) were used to determine the best trajectory model fit to the biomarkers and self‐report scores. Trajectory group membership was saved for subsequent plotting of the trajectory patterns and for assessing associations of hypothesized patient and treatment characteristics with those trajectories using likelihood ratio chi‐square and Kruskal‐Wallis tests. To capture natural trajectories for the 24‐month period, if a patient triggered the study intervention during the subclinical stage or progressed to treatment status without moving through the subclinical stage, assessments only up to and including the last assessment either triggering the prevention intervention or progressing through that trigger (TM: n = 79 of 245, BIS: n = 48 of 263), were included in the models. sas (9.4), Stata (13), and spss (22) were used for statistical analyses. A maximum Type I error rate of 0.05 (*P* < .05) was used for determining statistical significance. The data that support the findings will be available at ftp.impedimed.com following an embargo from the date of publication to allow for commercialization of research findings.^18^

## 3 RESULTS

### 3.1 Sample characteristics

Detailed patient and treatment characteristics for the 508 patients included in the analyses were previously published.^3^ Most patients were white (77%, n = 389). Slightly more than a third had Stage II/III disease (39.0%, n = 198). Summaries of the characteristics used in these analyses are shown in Table 1. No statistically significant differences between the groups were observed. Median age was 59 years (interquartile range, IQR = 50‐67), approximately 74% (n = 373 of 507) were either overweight or obese.

### 3.2 Trajectories

**Figure 1:**
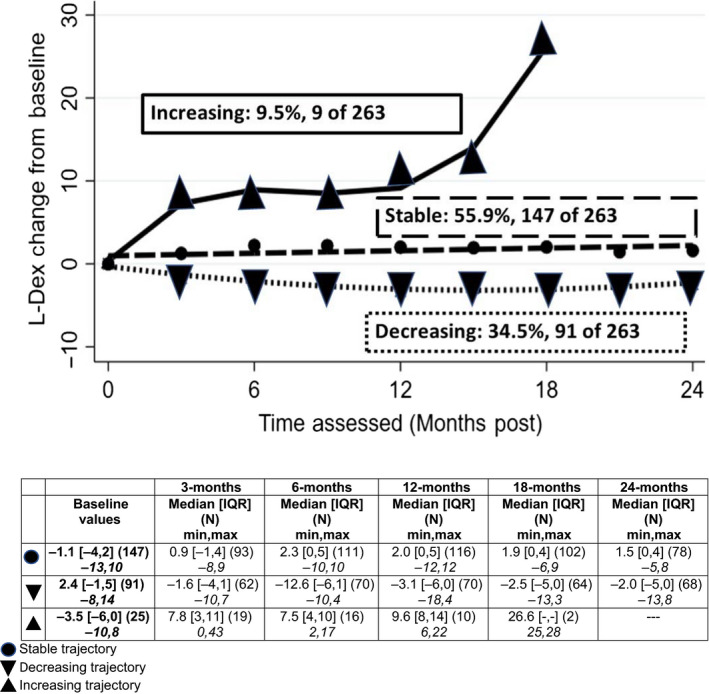
L‐Dex trajectories

**Figure 2:**
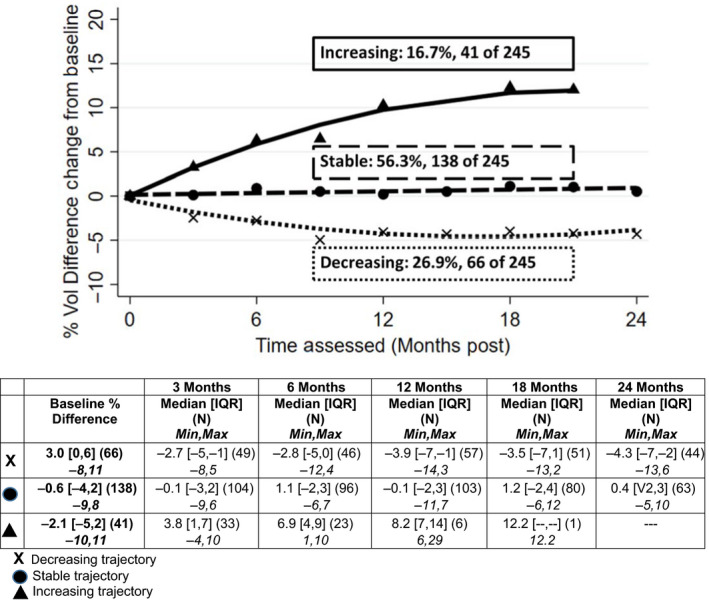
Volume trajectories

**Figure 3:**
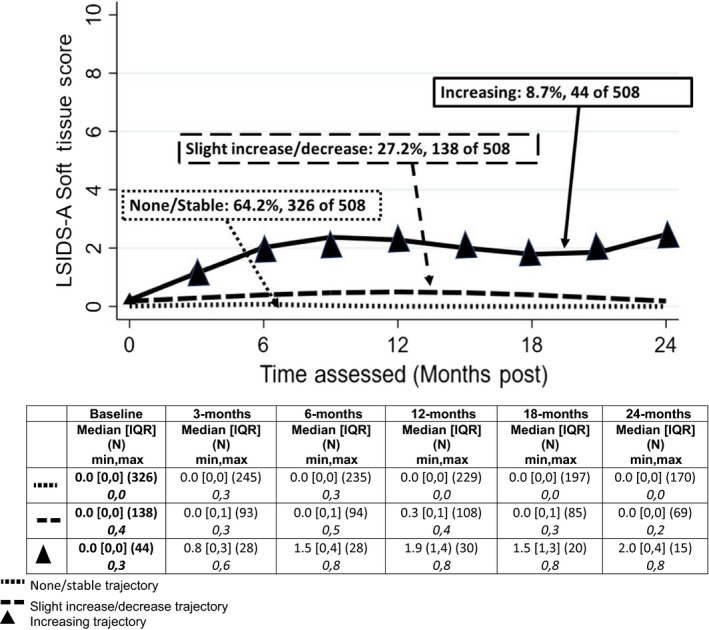
LSIDS‐a soft tissue symptom trajectories

**Figure 4:**
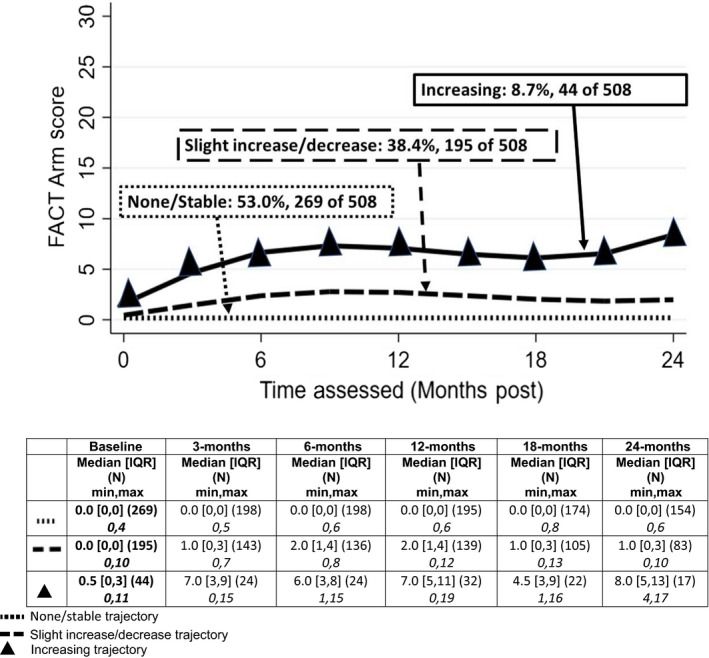
FACT B +4 arm symptom trajectories

Three trajectories were observed for each of the four measures: changes in L‐Dex obtained by BIS, percent volume difference change by TM, and symptom scores using LSIDS‐A and FACT‐B + 4. Each biomarker trajectory was categorized as “Decreasing”, “Stable” or “Increasing” over the 24 months. Symptom trajectories were characterized as “Stable”, “Slight Increase/Decrease” or “Increasing” as there was not a definitive decreasing trajectory. The patterns are illustrated in Figures 1, 2, 3, 4 and summarized below.

#### 3.2.1 L‐Dex

Slightly more than half (56%) demonstrated stable values from baseline through 24 months (Figure 1). Approximately a third demonstrated decreasing L‐Dex values (values lower than baseline, 35%), while 9% demonstrated patterns consistent with increasing L‐Dex values (n = 25 of 263). In the group with increasing L‐Dex values over time, increases were initially noted at approximately 3 months postsurgery, and at 15 months postsurgery, a second increase was noted (see Figure 1). Those patients in the decreasing L‐Dex values trajectory group had higher baseline L‐Dex values (median = 2.4, IQR = −1, 5) than did those in the other trajectory groups (stable: median = −1.1, IQR = −4, 2; increasing: median = −3.5, IQR = −6, 0, *P* < .001).

#### 3.2.2 Percent arm volume difference

Slightly more than half (56%) of the patients demonstrated stable arm volume difference, 27% demonstrated a pattern of decreasing difference between arm volumes, and 17% (n = 41 of 245) showed increasing arm volume difference (Figure 2). Increases were initially noted at approximately 3 months postsurgery with a steadily increasing slope over time. The percentage of patients assessed with TM, demonstrating the increasing trajectory pattern (17%), was statistically significantly higher than the percentage of those assessed via BIS with increasing trajectories (9%, *P* = .023). Similar to those patients randomized to the BIS assessments, patients with TM assessments, and in the decreasing percent arm volume difference trajectory, had higher baseline values (median = 3.0%, IQR = 0, 6) than did those in the other trajectories (stable: median = −0.6%, IQR = −4, 2; increasing: median = −2.1%, IQR = −5, 2, *P* < .001.

#### 3.2.3 LISDS—A soft tissue

Almost two‐thirds of the patients (64%) had stable soft tissue symptoms over 24 months (Figure 3). Another 27% had slightly increased symptom reports during the first 12 months after surgery that decreased to near presurgery level by 24 months. The remaining 9% (44 of 508) had an increasing trajectory characterized by steady rise in symptoms from baseline to 6 months postsurgery and remained well above baseline through 24 months postsurgery.

#### 3.2.4 FACTB+4

Slightly more than half of the patients (53%) had little to no symptoms throughout; 38% demonstrated a pattern of slightly increased symptoms the first 9‐12 months after surgery that stabilized and trended downward toward presurgery levels by 24 months (Figure 4). Finally, 9% (n = 44 of 508) demonstrated an increasing trajectory of symptoms from baseline to 12 months postsurgery and remained well above baseline through 24 months. Consistent with the similar patterns and proportions of patients in each pattern for the two self‐report measures, the contingency coefficient for the association was 0.59 (*P* < .001).

### 3.3 Associations among trajectories

The symptom trajectory groups were statistically significantly associated with distribution of L‐Dex change trajectories but not with percent difference change in arm volume trajectories. The self‐report sets demonstrated contingency coefficients of 0.20 (LSIDS‐A soft tissue, *P* = .031) and 0.19 (FACTB+4, *P* = .044) with the set of L‐Dex unit change trajectories. Of the 25 patients in the increasing L‐Dex change trajectory group, 24% (n = 6) were also in the increasing LSIDS‐A soft tissue trajectory and 16% (n = 4) in the increasing FACTB+4 trajectory. To the contrary, of the 41 in the increasing percent difference in arm volume change trajectory group, only 10% (n = 4) were also in the increasing LSIDS‐A soft tissue trajectory and 10% (n = 4) in the increasing FACTB+4 trajectory.

### 3.4 Patient and treatment characteristics with trajectories

Summaries of patient and treatment characteristics hypothesized to be associated with the longitudinal patterns of change in biomarkers and symptoms are shown in Tables 1 and 2.

#### 3.4.1 Bioelectric impedance spectroscopy

Patients in the increasing L‐Dex unit change pattern were older (median 64 years) than those in the stable or decreasing change patterns (median 59 and 58 years, respectively). They also were using nonsteroidal antiinflammatory drugs (NSAIDs) (48%) more than those in the stable trajectory group (20%). Patients in the decreasing L‐Dex trajectory were less likely to have a history of cardiovascular conditions (34%) than those in the stable group (50%) (all Bonferroni‐corrected, *P* < .05, Table 1).

#### 3.4.2 Tape

Patients in the increasing percent difference in arm volume trajectory were less likely to have a history of gastroesophageal reflux disease (GERD) (40%) than those in the stable group (84%, post hoc corrected *P* < .05, Table 1).

#### 3.4.3 Self‐reported symptoms

Patients with an increasing (slight or considerable) reported symptom trajectory were slightly younger than those in the group with very few reported symptoms throughout the observation period. They also were more likely to have had a history of respiratory issues (Table 2). In terms of treatment, compared to those patients with a null symptom trajectory, those characterized by an slight or increasing symptom trajectory were more likely to have axillary lymph node dissection only, regional node irradiation and a combined surgery/radiation/chemo therapy regimen with taxane and less likely to have had surgery/radiation alone (all post hoc corrected *P* < .05, Table 2).

## 4 DISCUSSION

This study provides longitudinal data regarding the postsurgical patterns of subclinical BCRL needed to inform prospective surveillance guidelines and clinical practice. One trajectory for each biomarker demonstrated slight decreases from baseline; in a second, there was little to no change from baseline measures. These two trajectories suggest that cancer treatment does not always lead to poorer arm status during the first 24 months. Subclinical lymphedema was identified in a third trajectory for both BIS and TM groups that continued to rise beyond 12 months after surgery. When looking at the characteristics of patients in the increasing trajectory groups for both BIS and TM, it appears that these cohorts include higher rates of regional nodal irradiation, tri‐modality therapy with taxanes, mastectomies, and patients with elevated Body mass index (BMI)’s as compared to decreasing and stable trajectories (Table 1).

The increasing trajectories for both biomarkers demonstrate that a subset of patients are at high‐risk of subclinical lymphedema during the first 24 months after surgery; however, due to censoring of participants when subclinical lymphedema was noted, patterns beyond the date of censoring are unknown for either biomarker. Previous trajectory work covering 12 months post operatively, using BIS ratios, identified three trajectories, all described as being “relatively stable” over 12 months and suggested BIS ratios 1 month postsurgery might predict lymphedema.^13^ In this study, spanning 24 months post surgery, only one stable trajectory was maintained over time. Of clinical importance is the trajectory increase noted in L‐Dex units at 15 months. Long‐term prospective surveillance is needed through at least 24 months postsurgery and frequent monitoring of patients every 3 months at least through 15 months postsurgery should be considered. Additionally, patients with characteristics similar to those noted in the third trajectories for both BIS and TM (eg, regional nodal irradiation, tri‐modality therapy with taxanes, mastectomies, and elevated BMI’s) should be encouraged to contact their providers between scheduled visits if they become symptomatic (new onset swelling or altered sensations in the arm).

Some patient/treatment factors were associated with trajectories for biomarkers and symptoms. Age, use of NSAIDS, and a history of prior cardiac conditions were associated with increasing L‐Dex trajectories, consistent with previous research.^19^, ^20^ GERD was associated with percent difference change in arm volume. No known studies support an association between GERD and BCRL. This is likely a spurious finding. Until a well‐tested, predictive model that identifies patients who absolutely fall into an increasing trajectory is proven accurate, prospective surveillance using objective biomarkers for all BCS is indicated.

Regardless of trajectory group, patients reported few to no baseline symptoms. Both symptom clusters were indicative of ongoing issues for about 10% of participants through 24 months after surgery. Being younger, respiratory health issues, type of complete treatment received, and regional nodal irradiation contributed to symptom severity. Younger BCS report more symptoms than do older survivors, therefore the age related findings are not surprising.^21^ Deep breathing improves lymph flow, thus respiratory conditions that constrain breathing might contribute to lymph stasis.^22^ Axillary lymph node dissection and radiation are known to be related to lymphedema, and additional research regarding the extent of surgery to the axilla +/− the extent of radiation to the regional nodes, L‐Dex increases, and symptoms is in preparation.^23^

This study found a second peak in increasing L‐Dex at 15 months. McDuff has previously shown that the hazard rate peak for lymphedema after axillary treatment varies by treatment complexity.^10^ In patients who received an axillary dissection alone, the hazard rate of lymphedema development was greatest in the first 6‐12 months. For patients who received axillary dissection with regional node irradiation, the hazard rate peaked between 18 and 24 months. Whereas in patients undergoing sentinel node biopsy with regional node irradiation, the hazard rate peaked between 36 and 48 months, presumably due to the slower onset of radiation induced changes such as fibrosis. Therefore, clinicians may wish to advise patients with these characteristics to contact them between visits.

Self‐reported symptoms were associated with L‐Dex changes, but not percent change in arm volume difference. Symptoms have long been considered warning signs in a subset of patients at risk for BCRL.^24^, ^25^ In 2017, the American Physical Therapists Association (APTA) recommended that: (a) self‐reported numbness, heaviness, etc. in at‐risk patients be assessed using an objective measure, and (b) BIS be used as a measurement tool for diagnosis of subclinical (stage 0) lymphedema.^26^ Symptom severity, from two symptom tools, was statistically significantly associated with L‐Dex changes indicative of subclinical lymphedema, but not with percent change in arm volume difference. This supports APTA’s recommendation that BIS be used for early identification of subclinical lymphedema, is consistent with previous research,^27^ and is clinically relevant for two primary reasons: First, the symptoms included in this study offer new information regarding symptoms associated with subclinical lymphedema. Second, BIS measurement captured early subclinical lymphedema when self‐reports of symptoms were present which provides evidence to support that BIS can identify subclinical lymphedema. Therefore, L‐Dex can be considered as a confirmatory biomarker for early identification of subclinical lymphedema, especially in the presence of patient‐reported symptoms.

## 5 CONCLUSIONS

New onset subclinical lymphedema was experienced by patients in the increasing trajectory across both biomarkers across 24 months postsurgery. These data support the need for prospective surveillance through at least 24 months postsurgery with 3‐month assessments in this population, through at least 15 months postsurgery. Statistically significant convergence of symptom cluster scores with L‐Dex unit change support BIS as beneficial in early identification of subclinical lymphedema.

## AUTHOR CONTRIBUTIONS

Conceptualization and study design—Ridner, Dietrich, & Shah. Data collection—Ridner, Boyages, Koelmeyer, Ajkay, DeSnyder, & McLaughlin. Statistical Analysis—Dietrich. Manuscript development and review: Ridner, Shah, Boyages, Koelmeyer, Ajkay, DeSnyder, McLaughlin, & Dietrich.

## Data Availability

The data that support the findings will be available at ftp.impedimed.com following an embargo from the date of publication to allow for commercialization of research findings.
